# Promoting healthy eating, active play and sustainability consciousness in early childhood curricula, addressing the Ben10™ problem: a randomised control trial

**DOI:** 10.1186/1471-2458-14-548

**Published:** 2014-06-03

**Authors:** Helen Skouteris, Susan Edwards, Leonie Rutherford, Amy Cutter-MacKenzie, Terry Huang, Amanda O’Connor

**Affiliations:** 1 School of Psychology, Faculty of Heath, Deakin University, Burwood, Australia; 2 Faculty of Education, Australian Catholic University, Banyo, Australia; 3 School of Communication & Creative Arts, Faculty of Arts & Education, Deakin University, Burwood, Australia; 4 School of Education, Southern Cross University, East Lismore, Australia; 5 Department of Health Promotion, Social, and Behavioral Health, University of Nebraska Medical Center College of Public Health, Omaha, Australia; 6 School of Psychology, Faculty of Heath, Deakin University, Burwood, Australia

## Abstract

**Background:**

This paper details the research protocol for a study funded by the Australian Research Council. An integrated approach towards helping young children respond to the significant pressures of ‘360 degree marketing’ on their food choices, levels of active play, and sustainability consciousness via the early childhood curriculum is lacking. The overall goal of this study is to evaluate the efficacy of curriculum interventions that educators design when using a pedagogical communication strategy on children’s knowledge about healthy eating, active play and the sustainability consequences of their toy food and toy selections.

**Methods/Design:**

This cluster-randomised trial will be conducted with 300, 4 to 5 year-old children attending pre-school. Early childhood educators will develop a curriculum intervention using a pedagogical communication strategy that integrates content knowledge about healthy eating, active play and sustainability consciousness and deliver this to their pre-school class. Children will be interviewed about their knowledge of healthy eating, active play and the sustainability consequences of their food and toy selections. Parents will complete an Eating and Physical Activity Questionnaire rating their children’s food preferences, digital media viewing and physical activity habits. All measures will be administered at baseline, the end of the intervention and 6 months post intervention. Informed consent will be obtained from all parents and the pre-school classes will be allocated randomly to the intervention or wait-list control group.

**Discussion:**

This study is the first to utilise an integrated pedagogical communication strategy developed specifically for early childhood educators focusing on children’s healthy eating, active play, and sustainability consciousness. The significance of the early childhood period, for young children’s learning about healthy eating, active play and sustainability, is now unquestioned. The specific teaching and learning practices used by early childhood educators, as part of the intervention program, will incorporate a sociocultural perspective on learning; this perspective emphasises building on the play interests of children, that are experienced within the family and home context, as a basis for curriculum provision.

**Trial Registration:**

Australian New Zealand Clinical Trials Registry ACTRN12614000363684: Date registered: 07/04/2014

## Background

‘360 degree marketing’ is the term given to the media environment that sees young children exposed to multiple forms of advertising for high calorie foods and consumable toys, clothing and products [1]. This term describes current marketing communication strategies through which children are targeted via multiple media, including radio, TV and social media, food packaging, point of sale advertising, direct mail, and blanket coverage of prime sites in public spaces. Traditionally, the impact of ‘360 degree marketing’ on young children is studied in diverse fields of research. For example, obesity research considers the influence of advertising on children’s food choices and the role of media viewing on children’s levels of physical activity [2,3]. Media research canvasses the types of media young children consume, the range of technologies they use to watch media, and the number of hours they typically spend engaged with media [4]. Environmental education examines the extent to which children’s digitally mediated experiences influence the quality of children’s active outdoor play, affinity with nature and children’s consciousness of the sustainability consequences associated with their clothing and toy preferences [5]. Meanwhile in early childhood education and early primary/elementary school settings, educators increasingly bemoan the extent to which ‘360 degree marketing’ influences young children’s play, the type of foods they bring to their centres and the range of clothing and toys in which they are interested [6].

An important issue for educators attempting to respond to this problem is the lack of research conducted from an integrated perspective into the best way to respond to young children’s exposure to saturation marketing via the early childhood curriculum. An integrated systems approach is needed to incorporate these important aspects, which are influencing children’s education and health and well-being. Encouraging children to take a holistic approach and enabling them to understand the consequences of their actions provides them with skills in the future to best handle unanticipated consequences.

Current contexts for children’s development in contemporary industrialised societies have been described by some social commenters as ‘toxic’ [7] promoting the phenomena of ‘nature-deficit’ [8] and ‘ecological illiteracy’ [9] among young children. These beliefs stem from the rapid adoption of digital technologies in society more broadly [10], and are aligned with advances in marketing techniques to young children that interface children’s affective engagement with popular culture characters with their engagements with digital media [11]. Young children are now commonly described as using a broad range of technologies to access and watch digital media that both promote particular characters [12], and expose children to advertising for high calorie foods, toys and clothing that have significant ‘cradle to grave’ impact on the environment [6,13,14].

In addition, research shows that increased digital media viewing is associated with reduced physical activity and levels of active play [15]. The combination of increased dietary intake of high calorie foods and reduced active play has had a profound impact on children’s weight gain across Australia, and other developed countries. Obesity in early childhood is currently recognised as a significant concern and one that is associated with children’s self-esteem placing them at later risk of depression [16]. Meanwhile, education for sustainability has become increasingly important for young children [17,18]. This has involved encouraging children to participate in green activities, such as gardening and composting food [19]. However, a more critical perspective in which children are encouraged to think about the environmental consequences of their consumer behaviour in light of their food and toy choices is yet to feature in early childhood education [12].

Sociocultural accounts of children’s learning and development promote strong connections between children’s home and community experiences and the provision of learning activities in early childhood education. This perspective is recognised in the notion of ‘funds of knowledge’ [20], where it is argued that curricula should be derived from children’s home and family interests and developed by educators as contextual responses to children’s cultural experiences [21]. This means that curricula driven by children, educators and families are recognised in early childhood education as more likely to achieve learning outcomes than imposed interventions. In the past, obesity prevention research directed towards young children has neglected to account for this perspective on teaching and learning and has been directed by the researchers and hence the research program instead [22]. We have developed a pedagogical communication strategy, using a ‘funds of knowledge’ perspective that acknowledges children’s likely engagement with digital media as a form of cultural experience; the strategy was developed in close consultation with children, families, and educators. Utilising the play experiences children have in their homes and communities are explored in the early childhood classroom. Educators may have further exposure to educational opportunities by asking children why they like to play with specific characters. A popular character for preschool children currently is Ben 10™; children are exposed to Ben 10™ through various digital mediums, which are then reinforced through marketing of Ben 10™ products such as clothes, toys and food. Consequently, the strategy is designed to enable educators to use their understandings of children’s play interests (such as wanting to play Ben10™) as the basis of the curriculum interventions.

The design focus on identifying children’s knowledge about healthy eating, active play, and sustainability, therefore, rests on a conceptual framework that strongly aligns with existing teaching and learning processes in early childhood education.

In early childhood education, the release of the Australian Early Years Learning Framework (EYLF) in 2009 [23] saw a focus on children’s wellbeing in terms of healthy eating and some awareness of environmental education in terms of children’s engagement with the natural world included in the early childhood curriculum. However, an integrated approach towards helping young children respond to the significant pressures of ‘360 degree marketing’ [1] on their food choices, levels of active play, and sustainability consciousness via the early childhood curriculum is lacking. The present study takes a targeted approach to the Ben10™ problem by providing educators with a pedagogical communication strategy for integrating healthy eating, active play, and sustainability consciousness in the early childhood curriculum. Because the pedagogical communication strategy is aligned with the Australian EYLF educators are able to use it in order to develop curriculum interventions that integrate obesity prevention, digital media and sustainability education experiences for young children; this is in direct contrast to previous approaches to health and sustainability education in early childhood, which has tended to focus on these issues separately. In addition, our proposed research accords with the *Australian Curriculum: Health and Physical Education* that requires teachers to provide opportunities for children and adolescents to adopt lifelong healthy, active living strategies that empower them to enhance their own and others’ health and wellbeing in varied and changing contexts [24]. Hence, the overall goal of the current study is to conduct a cluster randomised trial to evaluate the efficacy of the curriculum interventions that educators design when using the pedagogical communication strategy on children’s knowledge about healthy eating, active play and the sustainability consequences of their toy food and toy selections.

## Methods/Design

### Overall study design

This study is a cluster-randomised trial with pre-school classes of 4 to 5 year-old children randomly allocated to either an intervention or control group and will be conducted and reported in line with CONSORT recommendations. Baseline assessment of study variables will occur prior to implementation of the intervention. Follow up assessments will take place immediately following and 6 months post completion of the intervention strategy. The intervention group children will receive the curriculum interventions that their educators develop. The wait-list control group children will receive ‘usual care’, that is, their educator’s usual teaching approaches. The project has been approved by the Victorian Department of Education and Early Childhood Development and has received ethics approval from the Deakin University, Australian Catholic University and Southern Cross University Human Research Ethics Committee (DHREC 2013–220, 2014 39 V, and ECN-14-001, respectively).

### Hypotheses

In comparison to the control group, at the completion of the intervention and at 6 months post intervention there will be a significantly greater proportion of children in the intervention group who will: (1) show significant improvements in their knowledge about healthy eating, active play and the sustainability consequences of their food and toy selections; (2) demonstrate greater consumption of fruit and vegetables, and a decrease in consumption of high fat, and energy dense snack foods, especially those that are pre-packaged and marketed with popular media characters; and (3) demonstrate greater increases in active play and decreases in time spent in sedentary behaviours, specifically digital media viewing.

### Participants

The participants will be 300, 4–5 year old children, who attend preschool/childcare, their educators, and their English-speaking parents. The children will be attending early learning settings in the year prior to school and attending the first year of primary/elementary school in the following year.

### Recruitment strategy

The study will consist of at least 30 of the 68 available preschools (from Early Childhood Services Management (ECMS), Victoria) and aims to retain a total of at least 300, 4–5 year old children, who attend preschool together with their English speaking parents (because the program is only offered in English); the children will be in their final year of preschool. With a likely quota of 10 participants per preschool, 30 preschools (15 matched pairs) are required, 15 randomized to each arm of the study. Our formative work indicated a potential 11% attrition rate. Making a prudent allowance for attrition of up to 20%, 360 4–5 year old children and their parents will therefore be recruited and the adjusted number of preschools per study arm is likely to be 18 (i.e. a total of 36 kindergartens in 18 pairs).

## Procedure

### The Teacher Intervention

Teachers participating in the intervention group will be provided with a copy of the pedagogical communication strategy and asked to develop a curriculum intervention that integrates content knowledge about healthy eating, active play and sustainability consciousness. They will then attend a professional learning session to orientate them to the project. This session will be hosted by the Chief Investigators. Each teacher’s curriculum intervention will be workshopped to ensure they have included the following components: 1) implementing the interventions over 1–2 days across a period of 3–4 weeks to build a solid portfolio of experiences and strong base for children’s discussions; 2) implementing the interventions in the morning when the children are most cognitively alert; 3) combining both whole group and small group experiences and discussions; 4) using ‘real world’ props/resources, such as cereal boxes, McDonald’s Happy Meal™ boxes and toys and merchandise; and 5) maintaining assessment records of children’s learning using Learning Stories [25], observation and documentation. Teachers will be provided with an ‘*Intervention Implementation Checklist’* and asked to record all components/elements associated with their curriculum interventions including: date, time, and duration of the intervention; the number of times the intervention or iterations of the intervention were implemented; Early Years Learning Framework Learning Outcomes aligned with the intervention; complete description of the intervention (including any resources/props used) and examples of any approaches to assessing children’s learning used in relation to the intervention. A booster session will be conducted 6 weeks after teachers implement their curriculum interventions whereby children will be exposed to an abridged version of the intervention.

### Wait-list control group

The wait-list control group children will receive ‘usual care’, that is, their usual teaching and learning experiences as designed by participating educators. Qualitative and quantitative measures will be administered at the same time points as the intervention group. The teachers and children in this group will be offered the intervention at 7 months post baseline assessment.

### Measures

At recruitment, parents will be asked to complete an informed consent form confirming their participation in the study. The following measures will be administered at baseline, the end of the intervention and 6 months post intervention.

## Primary outcome

### Children’s knowledge about healthy eating, active play and the sustainability consequences of their food and toy selections

Children will be tested using a semi-structured interview based on the interview protocol used by [26] with 3–5 year olds to assess young children's healthy eating and active play knowledge. This will also include items investigating children’s understanding of sustainability concepts, such as waste and concepts of reduce, reuse, recycle and refuse (7Rs) [27]. A Research Fellow will interview children in a quiet area of their classroom, in full view of the teacher; teachers will be briefed to refrain from discussing which group they are in when the Research Fellow visits to ensure the Research Fellow is blind to randomisation.

## Secondary outcomes

### Children’s food preferences, digital media viewing and physical activity habits

The Eating and Physical Activity Questionnaire (EPAQ) [28] that has been developed by Australian researchers and has been assessed against a 24-hour dietary recall in 90 parents of pre-schoolers and found to be valid and reliable [28]. This method of food recall has low participant burden (takes about 5–10 minutes to complete) and hence is both time and cost-efficient.

### Qualitative interviews

We will invite 6 teachers from the intervention group to take part in an interview to obtain qualitative data in relation to barriers associated with educator use of the pedagogical communication strategy. These interviews (conducted by the Research Fellow) will take place 6 months post intervention, and will inform us about the strengths of the curriculum interventions. Fifteen parents of children in the intervention group will also be invited to be interviewed at this point to examine their perspective on their children’s learning about healthy eating, active play and the sustainability consequences of their food and toy selections. All interviews will take place over the phone at a time that suits the teacher and parent.

### Anthropometry

Height and weight will be measured with a stadiometer and standardised digital scales, respectively, at all assessments for children. Children will be weighed each time wearing light clothing and no shoes. Height and weight measures will then be converted to a Body Mass Index (BMI, kg/m2) for each participant. Body Mass Index will be standardised for age and gender using BMI-for-age z-scores and change will be assessed using BMI z-score slope [29] following WHO recommendations for children this age [30].

### Power, sample size and retention

The primary outcomes for this intervention are children’s knowledge about healthy eating, active play and the sustainability consequences of their food and toy selections. However, we decided to power our sample for the secondary outcome of child eating because we wanted to evaluate the extended effect of delivering the curriculum interventions to preschool children and we could base our sample size calculations on Australian data that provide relevant parameter estimates from three studies in the Barwon-South Western region of Victoria (*n* = 950) with children aged 2–4 years of age [31]. Vegetable consumption will require the largest sample size to demonstrate change over time, compared to other dietary outcomes, such as sweetened drinks, packaged snacks (e.g., chips, muesli bars), confectionary/chocolate, cake/sweet biscuits, and fruit, based on sample size analyses. As there are no quantitative dietary recommendations for children less than 4 years old in Australia, we adopted a suggestion of a 25% increase in vegetable consumption as a minimum target [32]. Based on findings that 63% of pre-school children had ≤1 serve of vegetables per day, we assume that the intervention will have a 25% absolute effect, that is the proportion of children in the intervention group who only have ≤1 serve of vegetables per day will change from 63% to 43%. We calculated the sample size necessary to detect a clinically meaningful difference in vegetable consumption, significant at the 0.05 level, with a power of 0.8; this calculation resulted in a sample of 150 children in each group. Making a prudent allowance for attrition of 20% (based on our previous research with preschool children and their parents), the adjusted number per treatment condition is 180. A total of 360 4-year-old children will therefore be recruited. This sample size is also sufficient to detect medium effect sizes in both the primary and secondary measures with a power of .8 at α = 0.05 [33].

### Analyses

Baseline quantitative data (collected from parents) will be secured prior to treatment allocation, missing values will be scrutinized to check for non-random distribution and primary analyses will be executed twice: once using observed data, and once using multiple imputations under multivariate normal assumptions using methods given by [34], so that all participants will be analysed in their allocated condition. Analyses of variance (with corrections for potential clustering effects of individuals within childcare centres, [32]) will test the between group differences in the primary outcome measure (children’s knowledge about healthy eating, active play and the sustainability consequences of their food and toy selections; interview data will be quantified, after each child’s interview has been transcribed, for the analyses) and the secondary outcome measures (child eating and activity habits) at each assessment time point and across time points for the children. The teachers’ qualitative interviews 6 months post intervention will be analysed using elements of phenomenology and thematic content analysis [35-37]. A process evaluation will be undertaken to identify the key elements of the curriculum interventions that result in significant and positive shifts in children’s knowledge about healthy eating, active play and the sustainability consequences of their food and toy selections.

## Discussion

The preschool period has been identified as a significant period for children’s learning about healthy eating, active play and sustainability [38-40]. The curriculum interventions designed by teachers during this project will be context-driven and specific to the teaching and learning practices associated with early childhood education. This is critical because it aligns with a sociocultural informed perspective on early childhood education that promotes drawing on children’s ‘funds of knowledge’ [20] as basis for curriculum provision [21]. Evaluating the efficacy of these curriculum interventions will enable us to determine the teaching and learning processes that impact children’s knowledge about healthy eating, active play, and sustainability. These processes represent the outcomes that will advance the knowledge base because, to date, there is little to no description in pre-service and in-service teacher education of how to approach the development of integrated curriculum in the early years in terms of children’s healthy eating, active play, and sustainability consciousness. The identified processes and the curriculum interventions designed by teachers will therefore provide clear examples of how early childhood curriculum can in fact be responsive rather than reactive to the pressures of 360 degree marketing on young children’s healthy eating, active play and sustainability consciousness [6]. Accordingly, the project outcomes represent a response to research describing the significance of the early childhood period for young children’s learning about healthy eating, active play and sustainability.

## Competing interests

The authors declare that they have no competing interests.

## Authors’ contributions

Together, HS, SE, LR, ACM and TH wrote and designed the study subsequently funded by the Australian Research Council and modified it for publication. AO is the research assistant appointed to manage the collection of data. All authors read and approved the final manuscript.

## Pre-publication history

The pre-publication history for this paper can be accessed here:

http://www.biomedcentral.com/1471-2458/14/548/prepub
